# Identification and DUS Testing of Rice Varieties through Microsatellite Markers

**DOI:** 10.1155/2015/965073

**Published:** 2015-02-08

**Authors:** Ehsan Pourabed, Mohammad Reza Jazayeri Noushabadi, Seyed Hossein Jamali, Naser Moheb Alipour, Abbas Zareyan, Leila Sadeghi

**Affiliations:** ^1^Department of Agronomy and Plant Breeding, College of Agriculture, Miyaneh Branch, Islamic Azad University, Miyaneh 53158-36511, Iran; ^2^Seed & Plant Certification and Registration Institute (SPCRI), Collection Avenue, Nabovvat Boulevard, Karaj 31535-1516, Iran

## Abstract

Identification and registration of new rice varieties are very important to be free from environmental effects and using molecular markers that are more reliable. The objectives of this study were, first, the identification and distinction of 40 rice varieties consisting of local varieties of Iran, improved varieties, and IRRI varieties using PIC, and discriminating power, second, cluster analysis based on Dice similarity coefficient and UPGMA algorithm, and, third, determining the ability of microsatellite markers to separate varieties utilizing the best combination of markers. For this research, 12 microsatellite markers were used. In total, 83 polymorphic alleles (6.91 alleles per locus) were found. In addition, the variation of PIC was calculated from 0.52 to 0.9. The results of cluster analysis showed the complete discrimination of varieties from each other except for IR58025A and IR58025B. Moreover, cluster analysis could detect the most of the improved varieties from local varieties. Based on the best combination of markers analysis, five pair primers together have shown the same results of all markers for detection among all varieties. Considering the results of this research, we can propose that microsatellite markers can be used as a complementary tool for morphological characteristics in DUS tests.

## 1. Introduction

In order to introduce a new plant variety to the markets commercially, it is necessary to register a newly bred variety, which relies upon the results of DUS (distinctness, uniformity, and stability) tests; that is, for a new genotype to be registered as a commercial variety, it needs to be distinct (D) from all other released varieties, uniform (U), and stable (S) for morphological and other evaluated traits [1, 2]. Therefore, DUS test has been established to be the foundation of plant variety protection and also to identify a new variety from reference collection [3].

The new variety should pass legal examinations to be commercialized and receive the certificate for the plant breeder's rights, a part of which consists of DUS tests according to morphological characteristics.

The current system of DUS testing has come across several significant shortcomings. The varieties to be assessed are increasing in number where their variability reduces, and the reference collections are expanding because of their internationalization, both of which result in the dramatic increase in expenses associated with these methods. Moreover, the existing methods are time consuming, which have altogether led to more necessity for developing a substitutionary, less costly system. Thus, the studies on the use of molecular markers in DUS testing proving the expected capability of molecular markers have encouraged International Union for the Protection of New Varieties of Plants (UPOV) to contemplate the introduction of molecular markers to the DUS testing system. Nevertheless, before this decision could be made, there are several issues to be resolved.

DUS testing would benefit from the use as molecular markers that have been shown to be more rapid and cost effective in comparison with morphological traits. In several registration processes such as cultivar identification, molecular markers have been utilized successfully [4].

As a prominent concern, molecular markers were not accessible, which have been gradually settled over time. Primarily, studies were restricted to using the dominant type of markers [5, 6] where continuous development of simple sequence repeat (SSR) markers has recently resulted in the prevalence of mentioned markers [2, 3, 7].

Microsatellite markers have been characterized with multiallelic nature, codominance inheritance, and relative abundance as well as requiring small quantities of DNA for amplification [8] which have made these markers efficiently applicable in DUS test of rice varieties [9, 10]. UPOV has confirmed the application of SSR markers as one of the commonly practical molecular marker systems for the identification of plant varieties [11].

This marker was previously confirmed to be applied to the distinction between plant varieties as well as complementary features in DUS tests where microsatellites were used in DUS tests on pepper [3], canola [2], and corn [12].

In this study, microsatellite markers was intended to identify rice varieties. The efficiencies of SSR markers were evaluated as complementary tools for the distinction of these varieties.

## 2. Materials and Methods

### 2.1. Plant Materials and DNA Extraction

In this study, 40 rice varieties consist of 27 varieties created in Rice Research Institute of Iran (RRII) and 10 local varieties from three regions of Guilan, Mazandaran, and Isfahan in Iran, and three International Rice Research Institute (IRRI) varieties were used (Table 1). All varieties used for this study were from* indica* subspecies. Fifteen seeds of each variety were selected and then 5 g of young and healthy leaves was used for DNA extraction. The DNA was extracted using the CTAB method with minor modifications (increasing extraction buffer density in two times and replacing Mercaptoethanol (0.2 percent) with Dithiothreitol (30 mM)) [13].

### 2.2. Microsatellites Reaction

Twelve pairs of SSR primers (a pair of primers of each chromosome) were selected from the panel of 50 from Gramene database (http://gramene.org/markers/microsat/50_ssr.html) (Table 2).

It selected a pair of primers from a mitochondrial DNA sequence (*drrcms* marker) (Forward: 5′ ACCTTTGGGCGATGGTT 3′; Reverse: 5′ GGGTTTAGAGTCGCCAC 3′) to detect the impurities in CMS line (IR58025A) from its cognate isogenic maintainer line (IR58025B) which is a prerequisite to obtain pure seeds of hybrid rice as well [14].

PCR reaction was carried out in a total volume of 15 *μ*L containing 3 *µ*L (25 ng) of template DNA, 1 *µ*L (0.66 *µ*mol/L) primers, 1.5 *µ*L 10x PCR buffer, 1.5 *µ*L dNTPs (0.2 mmol/L), 1.2 *µ*L MgCl_2_ (two mmol/L), 0.2 *µ*L* Taq* DNA polymerase (0.6 U/15 *µ*L), and 5.6 *µ*L ddH_2_O. An initial denaturation period of five min at 94°C was followed by 35 cycles of 60 s at 94°C, 30 s at 56–66°C, 120 s at 72°C, and then five min at 72°C for final extension. After amplification, the PCR products were separated on 6% (w/v) polyacrylamide gel and visualized by silver staining [15].

### 2.3. Statistical Analysis

The frequency of microsatellite polymorphism was calculated based on presence (1) or absence (0) of common bands. The genetic similarity between varieties was calculated using the Dice coefficient [16], and a dendrogram showing the genetic relationship of the 40 varieties was constructed using the unweighted pair group method with the arithmetic mean (UPGMA) [17] features of the NTSYS_pc_ v2.02 statistical analysis package [18]:

(1) $$\begin{array}{c} {\text{GD}}_{\text{NL}}=\left[2\frac{N_{11}}{\left(2N_{11}+N_{10}+N_{01}\right)}\right]. \end{array}$$

Accordingly, *N*_11_ is the number of bands (alleles) in both individuals; *N*_00_ is the number of bands (alleles) absent in both individuals; *N*_10_ is the number of bands (alleles) in *i*, *N*_01_ is the number of bands (alleles) present in *j*, and *N* is the total number of all bands (alleles).

Effective number of alleles (*A*_*e*_) [19] in each SSR locus was calculated by the following formula:

(2) $$\begin{array}{c} A_{e}=\frac{1}{\sum P_{i}^{2}}, \end{array}$$

where *P*_*i*_ is the frequency of *i*th allele for each locus.

The polymorphic information content (PIC) [20] of microsatellite loci was calculated according to the following formula:

(3) $$\begin{array}{c} \text{PIC}=1-\overset{n}{\underset{j=1}{\sum }}P_{ij}^{2}, \end{array}$$

where *P*_*ij*_ is the frequency of the *j*th allele for *i*th marker and the summation extends over *n* alleles.

Moreover, the discriminative power of molecular markers (*D*_*j*_) and the best combination of microsatellite markers (*X*_*k*_) [21] were estimated using the following steps and formulas.

There are *N*(*N* − 1)/2 different pairs in a set of *N* individuals. Based on this, *c*_*i*_ is the *i*th pattern of the given *j*th primer in which formula is

(4) $$\begin{array}{c} c_{i}=p_{i}\frac{\left(Np_{i}-1\right)}{N-1}. \end{array}$$

Moreover, for the *j*th primer, *C*_*j*_ is equivalent to the summation of the different *c*_*i*_ for all *I* patterns generated by the primer:

(5) $$\begin{array}{c} C_{j}=\overset{I}{\underset{i=1}{\sum }}c_{i}=\overset{I}{\underset{i=1}{\sum }}p_{i}\frac{\left(N_{p_{i}}-1\right)}{N-1}. \end{array}$$

Therefore, the discriminating power of the *j*th primer and the best combination of *k* primers are equal to

(6) Dj1−Cj=1−∑i=1IPiNPi−1N−1,XkNN−12∏j=1kCj.

## 3. Results and Discussion

### 3.1. Evaluation of Microsatellite Markers

The 12 microsatellite primers used for this study generated totally 83 polymorphic fragments with an average of 6.91 alleles per locus. Among these markers, RM316 with 13 alleles and RM55 with three alleles had the highest and lowest variation, respectively. Effective number of alleles was calculated from 2.44 (RM161) to 9.52 (RM316) with an average of 4.90 per locus. Additionally, the PIC was estimated from 0.52 (RM161) to 0.9 (RM316) with an average of 0.74 per locus. The discriminating power (*D*_*j*_) ranged between 0.57 (RM161) and 0.93 (RM316) with an average of 0.78 per locus (Table 2). The most of microsatellite markers had a high PIC and discriminating power. However, a few markers had a low range of PIC and discriminating power such as RM55 and RM161. Our results agree partially with those of Hashemi et al. [22], who utilized 10 microsatellite markers to characterize the genetic diversity in a group of 16 Iranian rice hybrids. PIC is regarded as one of the important features of the molecular markers and can be used to evaluate the differentiation ability of the markers [23].

### 3.2. Genetic Similarity and Relationships among Varieties

**Figure 1:**
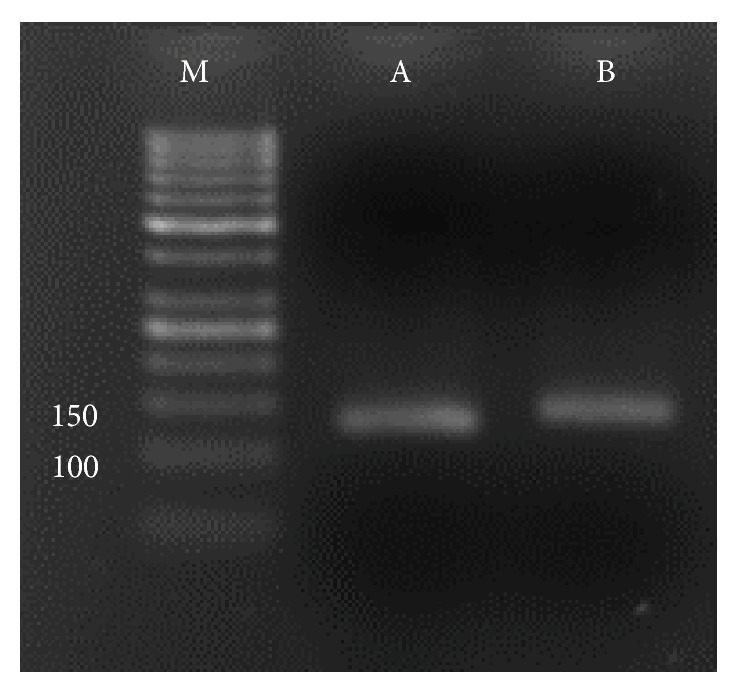
Detecting the impurities in CMS line from its cognate isogenic maintainer line. Polymorphism between CMS (IR58025A) and maintainer line (IR58025B) of rice at mitochondrial* drrcms* marker.

The range of similarity among varieties was from 0 to 1 with an average of 0.26 and variance of 0.063 for all microsatellite markers. Similarity values in between varieties were 0 for 66 pairs of varieties (supplementary file 1; see Supplementary Material available online at http://dx.doi.org/10.1155/2015/965073) and similarity value had been 1 just for one pair of varieties (IR58025A versus IR58025B). IR58025A is a CMS line, and, for detecting the CMS line from its cognate, isogenic maintainer line (IR58025B) was used; the polymorphism of a mitochondrial DNA sequence (*drrcms* marker) between some of CMS population and their fertile lines is verified in both lines (Figure 1).

**Figure 2:**
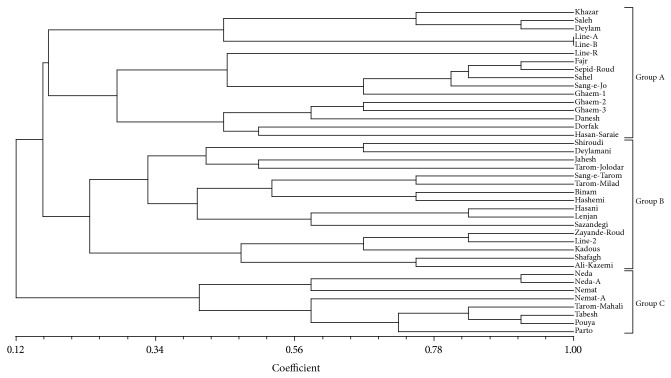
Dendrogram of 40 rice varieties based on UPGMA cluster analysis of Dice similarity matrix calculated from 12 microsatellite markers.

Dendrogram resulted from cluster analysis using UPGMA algorithm based on the Dice similarity coefficient and could discriminate all varieties from each other except for two isonuclear lines (IR58025A and IR58025B) (Figure 2). As a result, total microsatellite markers could detect most of the improved varieties (Group A) from local varieties (Group B). However, Sang-e-Jo and Hassan-Saraie as local varieties stood with improved varieties in Group A, because Sang-e-Jo and Sepid-Rood were used as recipient parents for Ghaem-1 variety. As can be observed in the dendrogram, Sang-e-Jo, Sepid-Rood, and Ghaem-1 have been in the same subcluster. Five improved varieties stood beside local varieties in Group B as well. These varieties are Shafagh and Kadous that improved from two IRRI lines as IR67015-94-2-3 and IR64669-153-2-3, respectively. In addition, Jahesh variety is a mutation of a local variety named Tarom. Sazandegi, purified from Lenjan local mass, and Shiroudi improved by cross between Deylamani as a local variety and Khazar as an improved variety. Consequently, Jahesh stood with other varieties obtained from Tarom such as Tarom-Jolodar, Sang-e-Tarom, and Tarom-Milad in Group B, and Sazandegi with Lenjan and Shiroudi with Deylamani constructed identical subcluster in Group B. There is an interesting result in the third group (Group C), in which three varieties, namely, Tabesh, Pouya, and Parto, constructed the same subcluster with Tarom-Mahali. These varieties are mutant of Tarom-Mahali.

Although the rice varieties in this study were from different rice breeding programs in Iran, microsatellite markers correctly grouped them depending on their respective group, local or improved varieties, in the dendrogram. Our results approve those of Garland et al. [24], who analyzed 43 rice samples using microsatellite markers and obtained a similar classification of varieties according to their breeding programs.

In addition, it obtained results from cluster analysis of each marker which have shown the ability of some of the markers as unique identification key for some of the varieties (Table 3).

### 3.3. The Best Combination of Markers

For finding the best combination of markers that result in the obtained result of using all markers in discrimination of all varieties, first, markers were chosen one after another in a way to minimize *X*_*k*_, that is, the number of pairs of distinct varieties for each primary compound in each step. In the first step, RM316 was chosen which distinguished the highest pairs of varieties from each other among *N*(*N* − 1)/2 pairs of varieties and made the amount of *D*_*j*_ maximum. In the second step, the compound of each *n* − 1 remaining marker with the chosen marker of previous step was tested in order to determine the most efficient compound that minimizes the amount of *X*_*k*_. In this step, compounding the RM271 with previous marker left the lower number of variety pairs undetermined. In subsequent steps, the same method was used for keeping or omitting other markers. Finally, adding RM154 to previous markers could decrease expected number of undetermined pairs of varieties calculated from 39.27 to 0.03 which should practically reach one pair of variety. There were 41 pairs of varieties nondistinct from each other in calculating similarity coefficient among varieties and then cluster analysis of varieties using RM316 that had the highest discriminating power (0.93) among markers. By adding consequent markers in accordance to distinguish the ability to this marker, the number of undistinguished pairs of varieties was decreased to one (Table 4).

Reliable identification of similar varieties is so difficult in plant species by morphological characteristics alone, because morphological and physiological characteristics are limited [25, 26]. Accordingly, using molecular markers as additional information is inevitable in registering plant varieties considering their benefits. DNA markers can be utilized to simply and rapidly detect varieties or approve the distinctiveness of a varietal impostor [27]. For identification and characterization of rice varieties and the testing of hybrid rice lines, using STS and SSR markers was significantly easier than using typical “grow-out tests” that included growing plants to maturity and evaluating purity based on morphological characteristics [28, 29]. Also, microsatellite markers have been utilized in the previous same study to distinguish traditional rice (*Oryza sativa* L.) varieties from each other in Cuba [30]. In this research, the results showed that microsatellite markers easily could be used for identification of rice varieties.

## Supplementary Material

Pairwise genetic similarity index of all varieties based on all of 12 microsatellite markers were used in this study.

## Figures and Tables

**Table 1:** Forty rice varieties used for this study, including name of variety and breeder.

Number	Variety name	Breeder
1	Parto	RRII
2	Jahesh	RRII
3	Nemat	RRII
4	Nemat-A	RRII
5	Neda	RRII
6	Neda-A	RRII
7	Tabesh	RRII
8	Sepid-Roud	RRII
9	Deylam	RRII
10	Sazandegi	RRII
11	Khazar	RRII
12	Danesh	RRII
13	Shafagh	RRII
14	Tarom-Jolodar	RRII
15	Saleh	RRII
16	Ghaem-1	RRII
17	Ghaem-2	RRII
18	Ghaem-3	RRII
19	Zayande-Rood	RRII
20	Sang-e-Tarom	RRII
21	Pouya	RRII
22	Line-2	RRII
23	Tarom-Milad	RRII
24	Dorfak	RRII
25	Sahel	RRII
26	Kadous	RRII
27	Fajr	RRII
28	Sang-e-Jo	Local
29	Shiroudi	Local
30	Hashemi	Local
31	Hassan-Saraie	Local
32	Ali-Kazemi	Local
33	Hassani	Local
34	Tarom-Mahali	Local
35	Lenjan	Local
36	Deylamani	Local
37	Binam	Local
38	IR58025A	IRRI
39	IR58025B	IRRI
40	IR42686R	IRRI

**Table 2:** Characteristics of polymorphic microsatellite markers used in this study, including locus name, number of chromosome, primer sequences, number of alleles, effective number of alleles, polymorphic information content (PIC), and discriminating power (*D*_*j*_).

Number	Locus name	Chromosome number	Primer sequences	Number of alleles	Effective number of alleles	PIC	*D*_*j*_
1	RM11	1	F: CAAATCCCGACTGCTGTCC R: TGGGAAGAGAGCACTACAGC	7	4.15	0.73	0.78

2	RM44	2	F: ACCCTCTCCGCCTCGCCTCCTC R: CTCCTCCTCCTGCGACCGCTCC	8	5.55	0.8	0.8

3	RM55	3	F: CCGTCGCCGTAGTAGAGAAG R: TCCCGGTTATTTTAAGGCG	3	2.73	0.6	0.68

4	RM124	4	F: ATCGTCTGCGTTGCGGCTGCTG R: CATGGATCACCGAGCTCCCCCC	8	5.81	0.8	0.83

5	RM133	5	F: TGCAGATGAGAAGCGGCGCCTC R: TGTGTCATCAGACGGCGCTCCG	4	3.16	0.63	0.73

6	RM154	6	F: TTGGATTGTTTTGCTGGCTCGC R: GGAACACGGGGTCGGAAGCGAC	6	5.32	0.79	0.82

7	RM161	7	F: TCTCCTCTTCCCCCGATC R: ATAGCGGGCGAGGCTTAG	4	2.44	0.52	0.57

8	RM237	8	F: ACGGGCAATCCGAACAACC R: TCGGGAAAACCTACCCTACC	5	4.63	0.75	0.8

9	RM271	9	F: CTAGTTGGGCATACGATGGC R: ACGCTTATATGTTACGTCAAC	10	6.58	0.83	0.88

10	RM277	10	F: TCAGATCTACAATTCCATCC R: TCGGTGAGACCTAGAGAGCC	7	4.6	0.76	0.8

11	RM287	11	F: TTCCCTGTTAAGAGAGAAATC R: GTGTATTTGGTGAAAGCAAC	8	4.4	0.74	0.78

12	RM316	12	F: CGGTCAAATCATCACCTGAC R: CAAGGCTTGCAAGGGAAG	13	9.52	0.9	0.93

Average				6.91	4.90	0.73	0.78

**Table 3:** Unique identification keys achieved by specific marker for some of the varieties.

Locus name	Varieties
RM44	Tarom-Mahali
RM124	Ali-Kazemi
RM11	Danesh and Sahel
RM271	Hassan-Saraie and Neda
RM316	Ghaem-1, Dorfak, Nemat, and Hassani
RM287	Khazar, IR42686R, Ghaem-3, and Sazandegi

**Table 4:** Comparison of combination of markers in the real and theoretical states under the hypothesis of independence of markers.

Locus name	Number of indistinguishable pairs	
	Experimentally observed	Expected under the independence hypothesis
RM316	41	39.27
RM316 + RM271	4	4.71
RM316 + RM271 + RM124	3	0.80
RM316 + RM271 + RM124 + RM154	2	0.14
RM316 + RM271 + RM124 + RM154 + RM44	1	0.03

## Abbreviations

DUS: Distinction, uniformity, and stability
PVP: Plant variety protection
PIC: Polymorphic information content
PCR: Polymerase chain reaction
SSR: Simple sequence repeat
UPGMA: Unweighted pair group method with arithmetic mean
UPOV: International union for the protection of new varieties of plants.

## References

[1] Lombard V., Baril C. P., Dubreuil P., Blouet F., Zhang D. (2000). Genetic relationships and fingerprinting of rapeseed cultivars by AFLP: consequences for varietal registration. *Crop Science*.

[2] Tommasini L., Batley J., Arnold G. M. (2003). The development of multiplex simple sequence repeat (SSR) markers to complement distinctness, uniformity and stability testing of rape (*Brassica napus* L.) varieties. *Theoretical and Applied Genetics*.

[3] Kwon Y.-S., Lee J.-M., Yi G.-B. (2005). Use of SSR markers to complement tests of distinctiveness, uniformity, and stability (DUS) of pepper (*Capsicum annuum* L.) varieties. *Molecules and Cells*.

[4] Mailer R. J., Scarth R., Fristensky B. (1994). Discrimination among cultivars of rapeseed (*Brassica napus* L.) using DNA polymorphisms amplified from arbitrary primers. *Theoretical and Applied Genetics*.

[5] de Riek J., Calsyn E., Everaert I., van Bockstaele E., de Loose M. (2001). AFLP based alternatives for the assessment of distinctness, uniformity and stability of sugar beet varieties. *Theoretical and Applied Genetics*.

[6] Law J. R., Donini P., Koebner R. M. D., Reeves J. C., Cooke R. J. (1998). DNA profiling and plant variety registration. III: the statistical assessment of distinctness in wheat using amplified fragment length polymorphisms. *Euphytica*.

[7] Cooke R. J., Bredemeijer G. M. M., Ganal M. W. (2003). Assessment of the uniformity of wheat and tomato varieties at DNA microsatellite loci. *Euphytica*.

[8] Becher S. A., Steinmetz K., Weising K. (2000). Microsatellites for cultivar identification in Pelargonium. *Theoretical and Applied Genetics*.

[9] Bonow S., von Pinho E. V. R., Vieira M. G. C., Vosman B. (2009). Microsatellite markers in and around rice genes: applications in variety identifi cation and dus testing. *Crop Science*.

[10] Singh R. K., Sharma R. K., Singh A. K. (2004). Suitability of mapped sequence tagged microsatellite site markers for establishing distinctness, uniformity and stability in aromatic rice. *Euphytica*.

[11] Upov-Bmt (2002). *MT/36/10 Progress Report of the 36th Session of the Technical Committee, the Technical Working Parties and Working Group on Biochemical and Molecular Techniques and DNA-Profiling in Particular*.

[12] Gunjaca J., Buhinicek I., Jukic M. (2008). Discriminating maize inbred lines using molecular and DUS data. *Euphytica*.

[13] Saghai-Maroof M. A., Soliman K. M., Jorgensen R. A., Allard R. W. (1984). Ribosomal DNA spacer-length polymorphisms in barley: mendelian inheritance, chromosomal location, and population dynamics. *Proceedings of the National Academy of Sciences of the United States of America*.

[14] Rajendrakumar P., Biswal A. K., Balachandran S. M., Ramesha M. S., Viraktamath B. C., Sundaram R. M. (2007). A mitochondrial repeat specific marker for distinguishing wild abortive type cytoplasmic male sterile rice lines from their cognate isogenic maintainer lines. *Crop Science*.

[15] Promega (2004). *SILVER SEQUENCE, DNA Sequencing System*.

[16] Nei M., Li W. H. (1979). Mathematical model for studying genetic variation in terms of restriction endonucleases. *Proceedings of the National Academy of Sciences of the United States of America*.

[17] Sneath P. H., Sokal R. R. (1973). *Numerical Taxonomy: The Principles and Practice of Numerical Classification*.

[18] Rohlf F. (1998). *Taxonomy, NTSYS-pc Numerical, Multivariate Analysis System*.

[19] Hartl D. L., Clark A. G. (1997). *Principles of Population Genetics*.

[20] Powell W., Morgante M., Andre C. (1996). The comparison of RFLP, RAPD, AFLP and SSR (microsatellite) markers for germplasm analysis. *Molecular Breeding*.

[21] Tessier C., David J., This P., Boursiquot J. M., Charrier A. (1999). Optimization of the choice of molecular markers for varietal identification in *Vitis vinifera* L.. *Theoretical and Applied Genetics*.

[22] Hashemi S. H., Mirmohammadi-Maibody S. A. M., Nematzadeh G. A., Arzani A. (2009). Identification of rice hybrids using microsatellite and RAPD markers. *African Journal of Biotechnology*.

[23] Ni J., Colowit P. M., Mackill D. J. (2002). Evaluation of genetic diversity in rice subspecies using microsatellite markers. *Crop Science*.

[24] Garland S. H., Lewin L., Abedinia M., Henry R., Blakeney A. (1999). The use of microsatellite polymorphisms for the identification of Australian breeding lines of rice (*Oryza sativa* L.). *Euphytica*.

[25] Giancola S., Marcucci Poltri S., Lacaze P., Hopp H. E. (2002). Feasibility of integration of molecular markers and morphological descriptors in a real case study of a plant variety protection system for soybean. *Euphytica*.

[26] Rongwen J., Akkaya M. S., Bhagwat A. A., Lavi U., Cregan P. B. (1995). The use of microsatellite DNA markers for soybean genotype identification. *Theoretical and Applied Genetics*.

[27] Collard B. C. Y., Vera Cruz C. M., McNally K. L., Virk P. S., Mackill D. J. (2008). Rice molecular breeding laboratories in the genomics era: Current status and future considerations. *International Journal of Plant Genomics*.

[28] Xu Y. (2004). Developing marker-assisted selection strategies for breeding hybrid rice. *Plant Breeding Reviews*.

[29] Yashitolaa J., Thirumuruganb T., Sundaramb R. M. (2002). Assessment of purity of rice hybrids using microsatellite and STS markers. *Crop Science*.

[30] Alvarez A., Fuentes J. L., Puldón V. (2007). Genetic diversity analysis of Cuban traditional rice (*Oryza sativa* L.) varieties based on microsatellite markers. *Genetics and Molecular Biology*.

